# The People of the State of New-York, vs. Wm. B. Waterman

**Published:** 1846-01-01

**Authors:** 


*The People of the State of New York vs. William B. Waterman, M. D.*—Some two months since, the defendant, in the cause entitled as above, a young physician who had shortly before opened an office in this city, was arrested on the charge of being engaged in raising bodies for anatomical purposes. Not being able to find bail he was committed to jail, and remained there until his trial, which took place on the 12th ult. He was found guilty of the offence, and was sentenced by the Court to the Auburn State Prison for 3 years. We are not personally acquainted with Dr. W., but we understand that ample testimony was produced on his trial of correct moral habits, industry, and promising attainments.

This is a hard case, and we deeply regret that the Court should have thought the circumstances demanded so severe a penalty. The regret and sympathy which have been excited do not appear to be confined to the medical profession, but are freely expressed by all the intelligent of this community whom we have heard speak on the subject.

Let us look for one moment at the position in which this sentence places the people of the State of New York. The people, it is to be presumed, have some interest in the possession of anatomical knowledge by those to whom the great majority trust their lives in cases of disease or accident. At all events, they require under heavy penalties the possession of this knowledge. But a young man is discovered in procuring the only means by which this knowledge is to be obtained. Who are his accusers? It is not contended that the feelings of surviving friends have been wounded. We have not heard that any complaints were made from this source, or that there were any friends to feel aggrieved. The informer was a miserable vagabond who was hired as an accomplice. The people thereupon become his accusers, and the majesty of the law is to be vindicated: for what?—for procuring the only means of obtaining that knowledge which is for the express purpose of benefitting the accusers, and which they, through legislation, have required, under penalties, at his hands. Constructively, it is not too much to say that this young man committed the offence by the express injunctions of the very power which, having detected him in the act, now sentences him to companionship with thieves, burglars, and villains of the deepest dye. The ends of his offence against the law were public benefit, and compliance with the requisitions of the law; and in return, the law immures him for three years in prison! Truly this is admirable consistency and justice for an enlightened government in this advanced period of civilization! The pacha of Egypt, notwithstanding the prejudices of heathenism and semi-barbarism, openly sanctions dissection, but the State of New York, while she demands the *results*, makes the conditions felony and ignominious punishment if discovery take place! So much for the abstract merits of the case; but what is the general practice incident to such a state of things? Why, every medical student and practitioner in the State of New-York is, or ought to be, directly or indirectly, guilty of this same species of felony. Dead bodies are stolen, and must be stolen; and all who study anatomy by means of them become *particeps criminis* in the theft. So far from wishing to have this fact concealed, if it be not generally so understood, we say let it be known. Let the community understand the subject in its full measure of injustice and absurdity. Let it be seen, that, as the law now stands, it exemplifies the principle of the Spartan code which made theft honorable, and the detection alone deserving of punishment. Would the community be satisfied if the medical profession were to unite in a determination to practice no more dissections? We trow not. Let the subject be viewed in its true light, and we cannot but believe that the present disgraceful condition of things will not be permitted to continue; the study of anatomy will be legalized, and ample facilities provided by law. In the mean time, we believe that public sentiment would sustain a jury that refused to convict for an offence of this kind, excepting when the feelings of friends were outraged; and that a judge would receive the thanks of the more intelligent of any community, who, upon conviction, would inflict a nominal punishment only, or, at least, a punishment which would not identify with crimes committed against the welfare of society, an act, the final purpose of which is not only salutary, but necessary.

